# Qualitative Exploration of Speech Pathologists' Experiences and Priorities for Aphasia Service Design: Initial Stage of an Experience‐Based Co‐Design Project to Improve Aphasia Services

**DOI:** 10.1111/hex.14105

**Published:** 2024-06-16

**Authors:** Lisa Anemaat, Victoria J. Palmer, David A. Copland, Geoffrey Binge, Kent Druery, Julia Druery, Kathryn Mainstone, Bruce Aisthorpe, Penelope Mainstone, Sarah J. Wallace

**Affiliations:** ^1^ School of Health and Rehabilitation Sciences, Queensland Aphasia Research Centre The University of Queensland Herston Australia; ^2^ Surgical Treatment and Rehabilitation Service (STARS) Education and Research Alliance The University of Queensland and Metro North Health Australia; ^3^ The ALIVE National Centre for Mental Health Research Translation The University of Melbourne Melbourne Australia

**Keywords:** aphasia, co‐design, coproduction, patient‐centred care, qualitative, stroke

## Abstract

**Introduction and Aims:**

Stroke survivors with aphasia (impaired language/communication) have poor outcomes and gaps in the clinical implementation of best practice contribute to this. Little is known, however, about speech pathologist perspectives on the touchpoints (key moments shaping experiences) in the clinical care pathway that have the greatest impact on service delivery nor how this varies by geographical location. We explored the experiences of speech pathologists who provide aphasia services to establish priorities for improvement and design.

**Methods and Analysis:**

This is the initial experience gathering and priority identification stage of an experience‐based co‐design (EBCD) project. Speech pathologists were recruited from 21 geographically diverse Hospital and Health Services in Queensland, Australia. Speech pathologists working in acute, rehabilitation and community services shared positive and negative experiences of delivering aphasia care in interviews and focus groups. Experiential data were analysed using qualitative thematic analysis to determine touchpoints. Priorities for service design were identified using an adapted nominal group technique.

**Results:**

Speech pathologists (*n* = 62) participated in 16 focus groups and nine interviews and shared 132 experiences of delivering aphasia care. Providing care in teams with poor awareness of the impacts of aphasia was identified as a key challenge, as poor patient‐provider communication was perceived to increase risk of adverse outcomes for patients. Speech pathologists identified areas for improvement related to their own professional needs (e.g., greater access to clinical supervision); collaborative health care (e.g., better coordination and interdisciplinary care to increase therapy time); and the service context and environment (e.g., psychological services able to support diverse communication needs).

**Conclusions:**

Speech pathologist delivery of aphasia services could be improved through increased access to clinical supervision, opportunities for peer debriefing and interdisciplinary care. Priorities for service design varied by geographical location and included: education to support care transitions (remote areas), improved referral pathways and service linkage (regional areas) and dedicated aphasia staffing (metropolitan areas).

**Patient or Public Contribution:**

A consumer advisory committee comprising people with aphasia (*n* = 3, authors K.M., K.D. and B.A.), their significant others (*n* = 2, authors J.D. and P.M.), and a Cultural Capability Officer (author G.B.) guided this research. The team: (1) reviewed participant information; (2) co‐designed surveys and workshop resources; (3) copresented research outcomes and contributed to publications. Research questions and study design (e.g., analysis methods and assessment measures) were developed by the research team (authors L.A., V.J.P., D.A.C. and S.J.W.).

## 1 Introduction

Aphasia is an acquired communication disorder that affects 30%–40% of stroke survivors [1] and can significantly decrease the quality of life [2]. People with aphasia experience greater social isolation [3], have long‐term negative outcomes [4, 5], and access more rehabilitation services than stroke‐survivors without aphasia [6]. Speech pathologists provide assessment and intervention to improve communication outcomes [7, 8]. However, variability in service models (e.g., workforce shortages, inflexible funding models) and clinical care pathways (e.g., evidence‐practice implementation gaps, restricted re‐entry to services) impact upon service provision and are known complications to managing care [9, 10, 11]. Improving outcomes for people with aphasia requires a context‐specific understanding of speech pathologists' experiences of service provision.

### 1.1 Complexities Delivering Care

In Australia, variations between local service contexts (e.g., time, staff availability, service guidelines, skilled supervision) impact on care management [12], limiting patient outcomes aligned with delivering recommended practice [13, 14, 15]. Allied health services play a critical role in improving health outcomes, particularly where smaller population densities and vast distances create inequities in service availability and access in remote communities [16]. Furthermore, speech pathologists are often required to prioritise the management of dysphagia (impaired swallowing) over aphasia, particularly in acute settings [15], reducing clinical capacity to provide recommended care. A finding that has more recently been echoed internationally, in a national survey of speech pathologists in Ireland [17]. These complexities present challenges when designing services able to improve care.

### 1.2 Meeting Patient Expectations

Clinician burnout can occur when gaps exist between patient expectations (of care provision) and service capacity to deliver care [18]. Gaps between service capacity (e.g., de‐prioritisation of aphasia care, expertise, time) and patient expectations (e.g., care in‐line with recommendations) may therefore leave speech pathologists who deliver aphasia care vulnerable to burnout, and contribute to poorer care experiences and health outcomes for patients. This makes understanding speech pathologist perspectives and experiences across service settings and geographic remoteness important for the design of services that can better meet the care recipient's needs.

### 1.3 Aims

Experience‐based co‐design (EBCD) [19], is a tripartite co‐design approach that brings together staff perspectives and priorities with patients and family to identify touchpoints (key moments shaping positive/negative experiences) and explore service design needs. As part of a larger EBCD study to improve aphasia services, speech pathologists were engaged to explore the care delivery journey, negative touchpoints impacting current aphasia care, and their priorities for service design. Experiences, priorities and touchpoints impacting patients (people with poststroke aphasia) and their significant others, collected as part of the larger study will be reported elsewhere.

## 2 Methods

### 2.1 Patient and Public Involvement

A consumer advisory committee comprising people with aphasia (*n* = 3, authors K.M., K.D. and B.A.), their significant others (*n* = 2, authors J.D. and P.M.), and a Cultural Capability Officer (author G.B.) guided this research. The team: (1) reviewed participant information; (2) co‐designed surveys and workshop resources and (3) copresented research outcomes and contributed to publications. Research questions and study design (e.g., analysis methods, assessment measures) were developed by the research team (authors L.A., V.J.P., D.A.C. and S.J.W.).

### 2.2 Study Design

**Figure 1:**
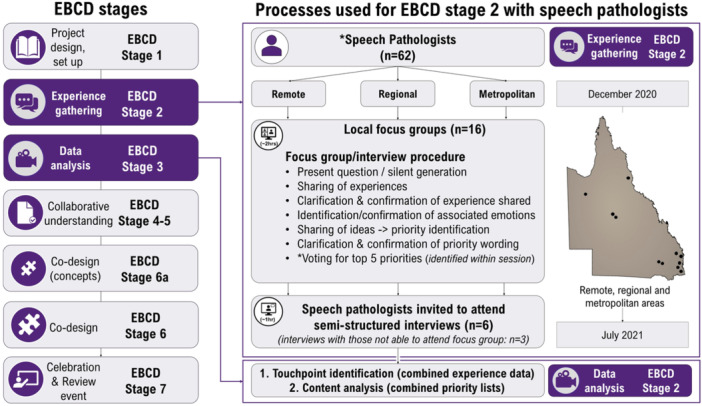
EBCD overview and EBCD‐2 processes. *Experience gathering stages were additionally conducted with people with post‐stroke aphasia (*n* ****= 32) and their significant others (*n* ****= 30), reported elsewhere. Within focus groups, ideas were transferred to an online survey before participants voting (to complete selections anonymously). Emotions were selected from a predefined list or self‐identification of an alternative.

**Figure 2:**
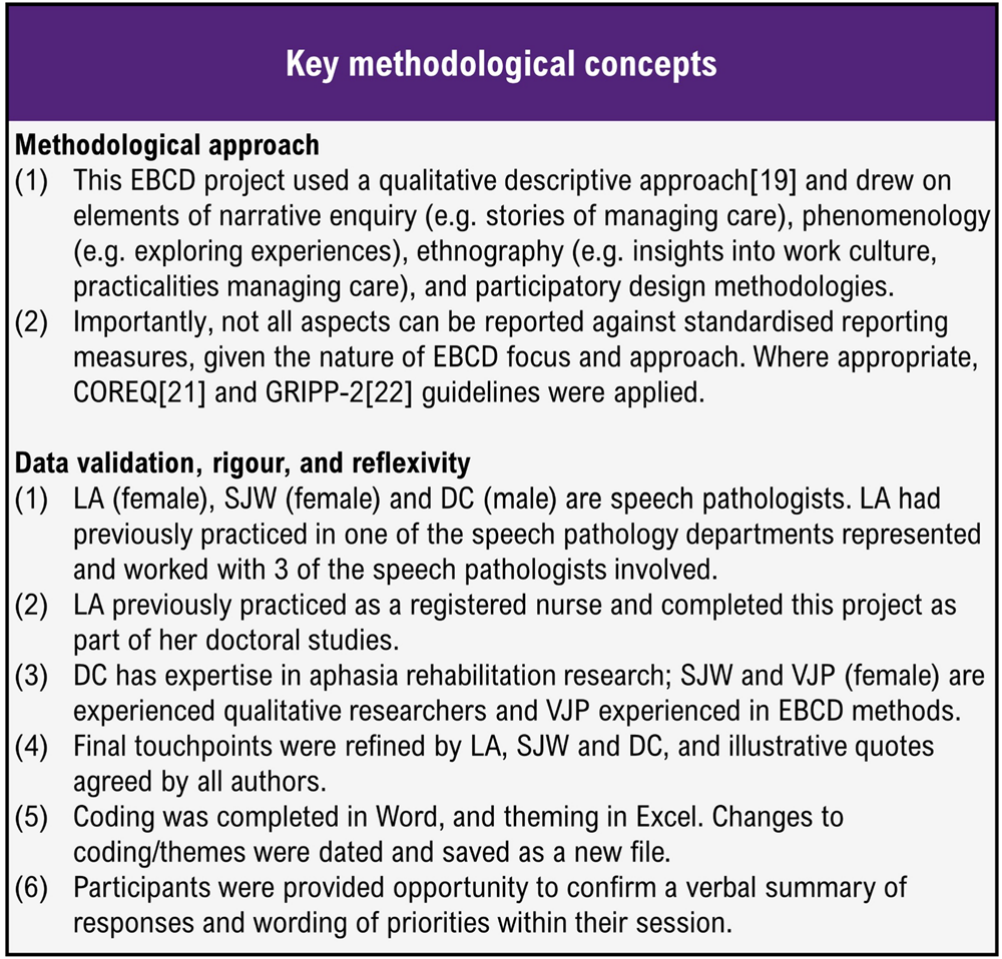
Key methodological concepts.

This paper reports the initial experience gathering and priority identification stages of an EBCD project [20] (Figure 1), focused on understanding experiences specific to speech pathologists to identify touchpoints (determining improvement areas from negative touchpoints). Experiences, touchpoints and priorities specific to people with aphasia and their significant others, gathered as part of the larger EBCD project are being reported elsewhere [21]. A qualitative descriptive approach [22] was used and a constructivist‐interpretivist paradigm guided analysis [23] (Figure 2). Detailed information about study design and sampling criteria are available in the published protocol [20].

### 2.3 Participants and Recruitment

Focus‐group participants were recruited from 21 hospital and healthcare sites (Supporting Information S1: SM1) and via online advertisements. Eligible participants were speech pathologists providing clinical services to people with aphasia in Queensland, Australia. Of those who consented, a subset (*n* = 6) [20] were purposively sampled, to complete in‐depth semi‐structured interviews.

### 2.4 Ethical Approvals and Patient Consent

Consent was obtained as per Royal Brisbane and Women's Hospital (HREC/2020/QRBW/61368) and The University of Queensland (2020000965) Human Research Ethics Committees. Where appropriate, COREQ [24] and GRIPP‐2 [25] guidelines were applied.

### 2.5 Data Collection

Data were collected between December 2020 and July 2021 using: (1) focus groups (≤5 speech pathologists per group) and (2) in‐depth semi‐structured interviews with a maximally varied sample (*n* = 6) and those unable to attend a focus group. Participants were sampled according to: (1) geographic remoteness; (2) years of clinical experience; and (3) type of health service (e.g., hospital‐based or community‐based rehabilitation service). Focus groups used an adapted nominal group technique [26] to explore experiences and establish priorities for service design. A deeper understanding of thoughts and actions associated with experiences were explored in interviews. An interview/focus‐group guide was developed comprising open‐ended questions [19]. Procedures and questions were piloted with speech pathologists (*n* = 5), resulting in modifications to wording clarity, processes for ranking and emotion identification. All interviews/focus groups were facilitated by L.A. (S.J.W. assisted with larger groups) and audio and video recorded. Sessions (interview/focus groups) were conducted online using video‐conferencing [27] during the COVID‐19 pandemic. Prioritisation of care during pandemic stages meant some clinicians were unable to attend a group session. These participants (*n* = 3) were provided the opportunity to share experiences and priorities individually.

#### 2.5.1 Procedures

Interviews/focus groups followed the same format (Figure 1): (1) sharing a positive and negative experience (managing care); (2) sharing ideas for aphasia service development. Ideas were transcribed onto an online whiteboard [27] (by author L.A.) as participants spoke, and modified until participants confirmed wording; (3) during interviews/focus groups participants then selected their top five ideas, ordered them from one (highest‐priority) to five (lowest‐priority), and allocated 100 points across selections (high number = higher importance) to indicate significance [28]. Feedback on processes and experiences of participation was collected (to be reported elsewhere).

### 2.6 Data Analysis

Experiential data from interviews/focus groups were transcribed and thematically analysed [29]. An inductive approach was undertaken to explore participants' experiences of delivering care and to understand implications for future service design. Themes were further reviewed for key moments eliciting a positive or negative response that shaped experiences for speech pathologists (touchpoints) [19]. Touchpoints (identifying key moments that shape experiences) were developed by considering the emotional and sensory connections described in experiences across the journey of care [30]. Themes of positive or negative experiences (and associated emotions), were mapped to three phases of care delivery: (1) hospital‐based care; (2) preparing for discharge or transferring to alternative service; and (3) community‐based care. Thematic analysis [29] was conducted by L.A. (S.J.W. reviewed all codes for consistency of interpretations). Improvement areas were determined by reviewing negative touchpoints for factors contributing to negative experiences per touchpoint. Participant priorities (combined lists from interviews/focus groups) for service design were analysed using qualitative content analysis [31] (completed by L.A., reviewed by S.J.W.).

Experience data captured per participant from both interviews and focus groups were combined, transcribed and individually coded. A verbal summary of experiences shared was confirmed within each session. Member checking involved summarising and reflecting participant responses during data collection to confirm meanings.

## 3 Results

Speech pathologists (*n* = 62) participated in focus groups (*n* = 16 groups; duration 74–142 min) and interviews (*n* = 9 interviews; duration 58–106 min) (Table 1). Five speech pathologists participated in both a focus group and interview. Technical issues resulted in two interview sessions not being recorded. Both speech pathologists were contacted to confirm a written summary of responses. Experience data for one participant could not be confirmed, and therefore experience data for one participant was not included in the final analysis, however, ranked priorities identified during both interviews were included. Illustrative quotes are presented with participant code and geographical remoteness classification. Interview data are indicated with an ‘a’ (e.g., SP‐001a‐remote).

**Table 1:** Participant characteristics (*n* = 62).

Characteristic	*n*	(%)
Geographic remoteness^a^		
Metropolitan	39	(63)
Regional	17	(27)
Remote	6	(10)
Years of experience working with people with aphasia		
<2 years	5	(8)
2–10 years	31	(50)
>10 years	26	(42)
Type of health service setting^b^		
Acute	12	(19)
Stroke‐unit	11	(18)
In‐patient rehabilitation	28	(45)
Out‐patient	20	(32)
Community‐based	17	(27)
Tele‐service	11	(18)
Other	4	(6)
Typical caseload (aphasia)‐self‐report		
Acute	18	(29)
Subacute	47	(76)
Chronic	21	(34)
Multiple	20	(32)

a Remoteness determined by postcode (Australian Bureau of Statistics Remoteness Area Classifications).

^b^ Speech pathologists worked across multiple settings.

### 3.1 Positive and Negative Touchpoints

**Figure 3:**
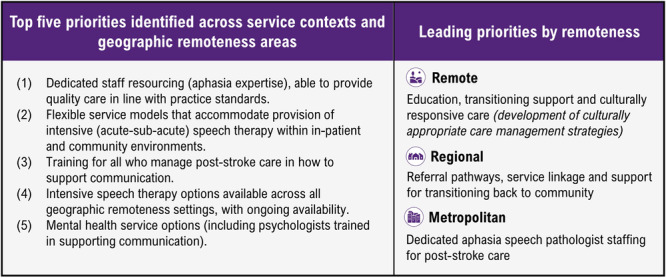
Top priorities identified.

Four touchpoints were identified from positive and negative experiences (Figure 3): (1) *service and organisational factors influencing quality of care delivered;* (2) *clinician factors impacting internal resilience and experiences of care delivery;* (3) *patient factors influencing care management, delivery and outcomes;* and (4) *external and environmental factors impacting care,* with two cross‐cutting themes: (1) *de‐prioritisation of aphasia care* and (2) *inequalities in accessing care* identified (Table 2). Across remoteness areas, collaborative interdisciplinary care was perceived to improve outcomes through ‘constant exposure, to all of the rehab goals’ (SP‐023‐metropolitan). Reporting focuses on highlighting negative themes, which will inform co‐design objectives in future stages (Supporting Information S1: SM2—Additional data).

**Table 2:** Overview of touchpoints relating to experiences with example quotes.

	Subthemes related to touchpoint	Example improvement	Example quotes within touchpoint
**1.** ***Service & organisational factors***	1.1. Ability to deliver comprehensive care: *Having your hands tied* 1.2. Personalising care & transition support: *There's nowhere to go* 1.3. Working in teams: Collaborative approaches to providing care 1.4. Poor awareness of the impact of aphasia	Lack time to reach goals or deliver dose Inability to deliver evidence‐based practice Not having service options to refer on to Not being able to manage procedural safety	…it's challenging to let them go‐ what I would feel a bit early, […] but then having to sell that to the patients. […] we obviously can't go in and sort of say, ‘I'd keep you, but no one else will’. (SP‐009‐metropolitan) …if somebody has to cut a session short, we don't have the ability to go, ‘right, well I'll try again later today’. (SP‐029‐metropolitan) I think what we offer is fraud actually. […] To tell people that what they're getting is evidence‐based practice when they're getting half an hour with a speechie twice a week in an inpatient setting. (SP‐030‐regional) We are a state‐wide rehab service, with rehab‐trained clinicians[…]still, our recommendations for how to communicate with aphasic patients definitely falls by the wayside. […]she came back with half‐done scans, and they had to go again. This lady was radiated twice[…] because[…] [nobody was] there to support her. (SP‐101‐metropolitan)
**2.** ***Clinician factors***	2.1. Emotional toll for clinicians: *I dreaded coming to work* 2.2. Clinical competence & professional support: *Trying to open a bank safe with a wet noodle* 2.3. Knowing care delivered was meaningful & had a positive impact	Lack debriefing opportunities (complex cases) Lack access to supervision	…very scared of her [person with severe aphasia, reduced insight, impulsive behaviours] […] sometimes I really dreaded, kind of, coming to work. (SP‐040‐metropolitan) …that rollercoaster ride where you're feeling good and they're feeling good and making plans for the future and then something happens‐ […] and we're back to square one again. (SP‐024‐metropolitan) …tricky emotionally managing the emotions of the team, who are also, very upset[…] you get to know their little kids. […] next minute you're having to discuss she's probably not safe to be left with the kids. (SP‐018‐metropolitan) I felt like I let that family down […] Frustrated for the patient, frustrated for ourselves as a profession, feeling like, you know, something's there, that the patient could use and maybe could benefit from […] not being skilled enough to go, ‘Hey there's this device!’. (SP‐048‐metropolitan)
**3.** ***Patient factors***	3.1. When PWA have good outcomes & communication successes 3.2. Patient engagement & motivation 3.3. PWA feel heard & are acknowledged 3.4. Aphasia severity or mobility restricts access to care 3.5. Managing emotional & mental health changes: *Grief is really hard*	Lack of psychology expertise within team	…someone who perhaps isn't willing to engage right now […] [not being able to reconnect with services] down the track. (SP‐060‐metropolitan) …helpless feeling at the thought of not only, ‘I've got skills to offer you, but I'm unable to offer them’. (SP‐029‐metropolitan) I'd say most of my caseload, would describe that they have low mood as a‐ as a result of their communication difficulties […] there was a patient who tried to access a psychologist and who was turned away […] because they didn't understand post‐stroke aphasia. […] it took her a lot to‐ to initiate that process, […] to not be able to‐ to receive it, because of the reason that she was accessing it[…]? How do you help? (SP‐041‐metropolitan)
**4.** ***External & environmental factors***	4.1. Family engagement: *It's not just the patient but support around them* 4.2. Cultural responsivity: No cultural support services in place 4.3. Aphasia research support or evidence practice gaps 4.4. COVID: Modification of care during a pandemic	Lacking resources to deliver culturally appropriate care Need upskilling in how culture impacts engagement Inadequate treatment options	… you know what the evidence is, and you know what you want to do and what you could do, and could achieve with this person, but you physically don't have the time or the capacity to be able to do that. (SP‐044‐remote) …there's more barriers to people who are culturally and linguistically diverse or, you know, in terms of even accessing interpreters to be able to do our job properly. Those sorts of things, like those really fundamental basic skills that that's not seen as a priority. (SP‐094‐metropolitan)

**Cross‐cutting themes**			
**Theme**	**Domain: Subdomain focus**	**Example improvement**	**Example quote**
**1.** ***De‐prioritisation of aphasia care***	**Context & environment**: Service environment **Collaborative care**: De‐prioritisation **Collaborative care**: Awareness of aphasia	MDT not valuing SP input into aphasia care Clinicians not able to deliver quality care in line with clinical guidelines PWA missing out on communication therapy while other treatments are prioritised	…people with aphasia, I feel, can often be deprioritized in the service […] these people with aphasia on the caseload may not receive the […] intense therapy that they require or level of support that they require […] the importance of‐ of the care […] particularly across other disciplines, um, so, allied health and medical, um, disciplines in particular, um, can sometimes have a lack of awareness of the impact of aphasia broadly. (SP‐031‐regional) …sometimes it feels like you're in a ballot. […] we want our patients to walk again, um, but at the same time, we want also our patients to be able to communicate. (SP‐036‐metropolitan) …it is really hard for other professions, to see how important it is, when our own profession doesn't prioritize aphasia and people with aphasia. (SP‐034‐regional)
**2.** ***Inequalities in accessing care***	**Context & environment**: Service environment **Context & environment**: Service options **Context & environment**: Treatment options	Lacking services in remote or regional areas Not having services to hand over care to	…access to aphasia services isn't always equitable and a lot of it does depend on the advocacy and the family support. Um, it also can even depend on the individual consultant who actually reviews the patient, um, and ‐ and that to be quite, I guess, distressing as a clinician when you're a lone voice kind of, um, you know, advocating for the person, and even though we know with global aphasia they may not make significant, you know, massive gains in terms of their communication, but there's so much work we can do with the family, there's so much work we can do in developing appropriate AAC support, even if it's about the person. (SP‐057‐ metropolitan) …it's hard, because you see the intensity‐ intensity for physio, we know it's required because we want our patients to walk again, um, but at the same time, we want also our patients to be able to communicate […] because, ultimately, if we can't provide them with that service, it's impacting on their recovery and their improvement, um, and at the end of the day, their quality of life. (SP‐036‐metropolitan)

Abbreviations: MDT, Multidisciplinary team; PWA, Person with aphasia; SP, Speech pathologist.

#### 3.1.1 Touchpoint 1. Service and Organisational Factors Influencing Quality of Care Delivered

Speech pathologists (across remoteness) reported that poor staff awareness of aphasia impacted service delivery. For example, lack of awareness of aphasia (as a sign of stroke) contributed to delayed diagnosis, or poor communication support during diagnostic imaging, delayed treatment. Others reported capacity to support patient transitions was impeded where follow‐on services were not available. In remote areas, maintaining continuity of care was influenced by clinical handovers, and participants reported feeling very frustrated when the ‘handover report that I got was just like a couple of lines’ (SP‐044‐remote). ‘Working in a rural generalist capacity’ (SP‐044‐remote) where ‘the lack of resources is a big thing’ (SP‐044‐remote), speech pathologists reported being unable to carry on care derived by specialist services.

#### 3.1.2 Touchpoint 2. Clinician Factors Impacting Internal Resilience and Experiences of Care Delivery

Speech pathologists often expressed feelings of guilt, sadness, or frustration associated with having *no choice* deciding when to end treatment (across remoteness). Supporting communication while expressing to patients the need to terminate treatment had long‐lasting emotional impact, particularly where this went against clinical decision making (e.g., best‐practice statement: ‘No one with aphasia should be discharged from services without some means of communicating needs and wishes’ [32]). Participants additionally described their emotional turmoil knowing when to begin therapy, and potential to cause harm if a patient was not ready. In metropolitan settings, feelings of inadequacy were expressed caring for those with severe impairments, or co‐occurring apraxia of speech, ‘I feel like I (pause) am trying to open a bank safe with a wet noodle’ (SP‐038‐metropolitan). In remote settings, participants expressed lacking confidence or skills to provide meaningful care regardless of seniority, ‘I felt so out of my depth, I had no idea what I was doing’ (SP‐045‐remote). These experiences stayed with participants. For generalist speech pathologists in remote areas, where clinical load extended the breadth of speech pathology practice, often as a sole practitioner—lack of supervision contributed to negative experiences: ‘Having a team to talk through that sort of stuff makes such a difference, […]by yourself and everything's overwhelming’ (SP‐045‐remote).

#### 3.1.3 Touchpoint 3. Patient Factors Influencing Care Management, Delivery and Outcomes

Patients emotional and mental health needs were reported to delay therapeutic engagement and limit time available to provide adequate aphasia rehabilitation, ‘medications didn't kick‐in in time and she was deemed inappropriate for rehab’ (SP‐072‐regional). Managing grief extended to supporting family, this was challenging where emotional recovery timelines were not aligned. Speech pathologists reported that *most* patients experienced ‘significant issues with their emotions[…][and not] having access to a psychologist’ (SP‐054‐metropolitan), put pressure on them to use therapy sessions providing this support. Both metropolitan and regional areas described instances where a patient did not ‘get the big time frame that we got with the other gentleman[…] because he was physically so able’ (SP‐072‐regional). Some expressed sadness managing care of those with severe global aphasia, particularly when they were denied intensive in‐patient rehabilitation, ‘they may not make significant, you know, massive gains in terms of their communication, but there's so much work we can do with the family’ (SP‐057‐metropolitan), or timing influenced access, ‘someone with global aphasia is actually more ready [for therapy] 4‐6 months down the track, and we're not actually able to provide therapy at that point’ (SP‐057‐metropolitan).

#### 3.1.4 Touchpoint 4. External and Environmental Factors Impacting Care

Speech pathologists observed that speaking a language other than English or being of Aboriginal and Torres Strait Islander origin was associated with poorer outcomes (all remoteness areas). Participants perceived this stemmed from a lack of suitable language assessments, therapy resources, research evidence, or time to adapt treatments, and felt frustration and guilt ‘That those patients miss out on a service that, yeah, that we have difficulty providing’ (SP‐014‐regional). Challenges with ‘health literacy’ (SP‐094‐metropolitan), supporting a patient to engage in therapy while navigating cultural differences or ‘the impact of shame [for some cultures]’ (SP‐094‐metropolitan) influenced care. Across remoteness areas, family involvement was perceived to increase patient engagement, enrich communication environments, improve outcomes and enhance access to care (e.g., transport or tele‐health support). Evidence‐practice gaps were frustrating for speech pathologists wanting to understand how best to support patients with milder deficits, a severe global impairment, or co‐occurring apraxia of speech. Those working in remote areas, where research evidence to inform care existed, also expressed frustration with knowing ‘what the evidence is, and you know what you want to do’ (SP‐044‐remote), but not being able to translate this into practice within their service context.

The unique circumstances of the COVID‐19 pandemic forced services to consider new, previously unconsidered models of care. Managing unpredictable changes to service delivery, or inability to deliver care were central to negative experiences. Despite these challenges, some described feeling ‘satisfied with the way that we were able to be flexible and deliver a service’ (SP‐008‐metropolitan), and felt care delivered was ‘a really good example of what we could offer’ (SP‐014‐regional). The perceived effectiveness of these ‘new models of care’, even for people with complex and severe case presentations, gave participants hope for what may be available in the future. Speech pathologists felt state‐wide collaborations between hospital service districts and university partners made this possible.

### 3.2 Cross‐Cutting Themes

1. **De‐prioritisation of aphasia care** influenced all areas. For some, this de‐prioritisation (prioritising swallowing management over aphasia) across the multidisciplinary team, was seen to directly contrast with their desire and capacity to deliver meaningful and comprehensive care, ‘it's not a personal decision but knowing that you could be providing care or support to these people with aphasia that we're just not able to provide’ (SP‐031‐regional). Speech pathologists expressed frustration with *de‐prioritisation from within the* [speech pathology] *team itself* (SP‐034‐regional) and commented that this internal de‐prioritisation made it ‘really hard for other professions to see how important it is’ (SP‐034‐regional). Knowing ‘you're sending these people out into the community without [*…*]their full intervention, their supports’ (SP‐034‐regional) was distressing.
2. **Inequalities in accessing care:** One participant reflected, access to ‘aphasia services isn't always equitable and a lot of it does depend on the advocacy and the family support’ (SP‐057‐metropolitan). Patient, service model, organisational and environmental factors all contributed to inequitable access to care and were frequently associated with feelings of frustration or distress for speech pathologists, ‘you feel like you can't do anything, you know?’ (SP‐096‐metropolitan). For people with aphasia discharged to residential care, speech pathologists reported, ‘you just don't have the same level of speech pathology service—often it's just dysphagia management’ (SP‐057‐metropolitan). Not having family involved in care or geographic remoteness impacted access, ‘if you're not living in a major capital city[…] then there's just no‐ no services in place’ (SP‐097‐metropolitan).

### 3.3 Local Priorities and Experience Map

**Figure 4:**
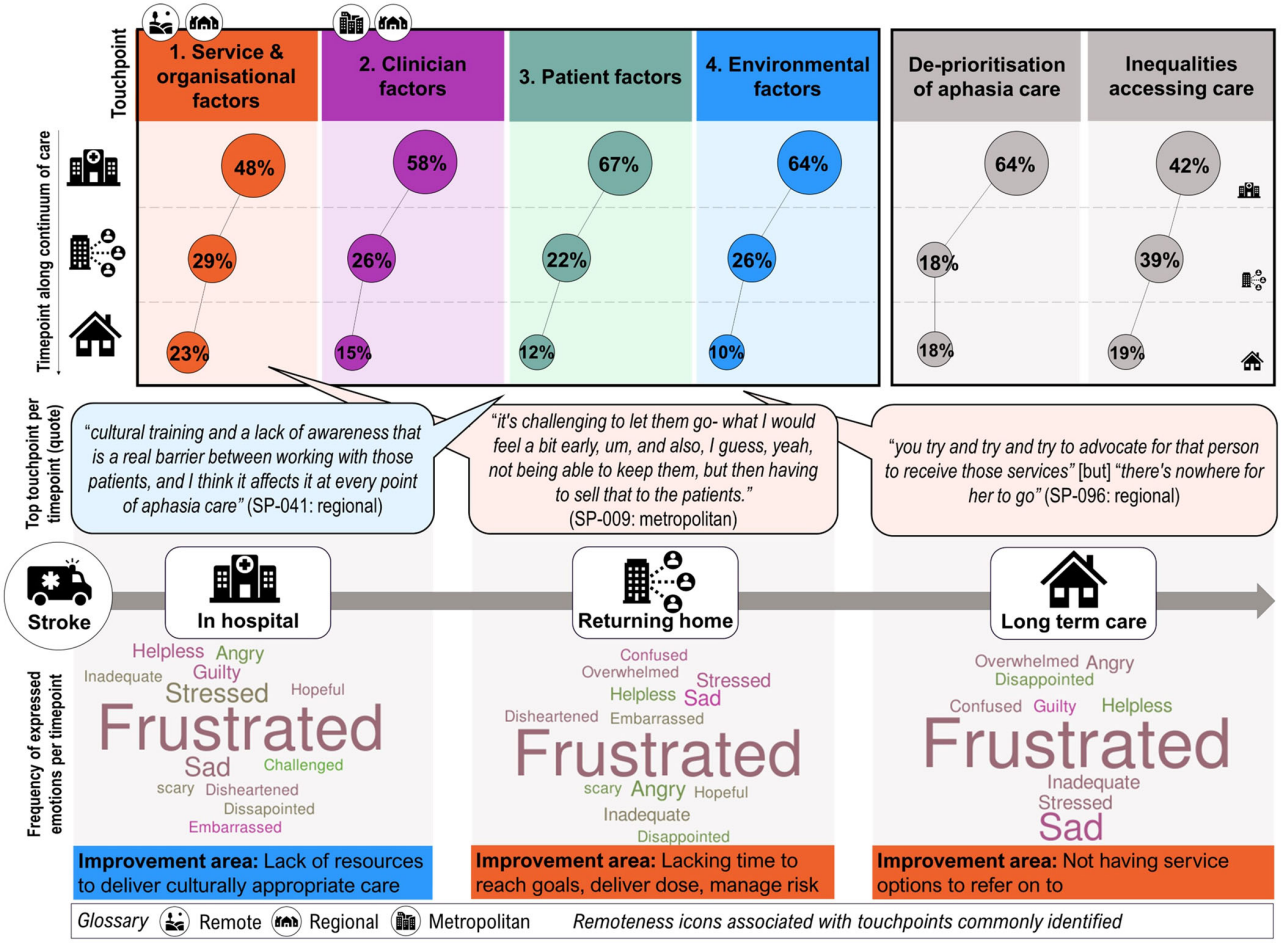
Experience map of negative touchpoints experienced by speech pathologists.

Speech pathologists generated 412 ideas for service design. These ideas were themed to produce 36 priority improvement areas. Top priorities (Figure 3 and Supporting Information S1: SM3), themes, and touchpoints are shown in the experience map (Figure 4).

### 3.4 Touchpoints Across Care Delivery Journey

**Figure 5:**
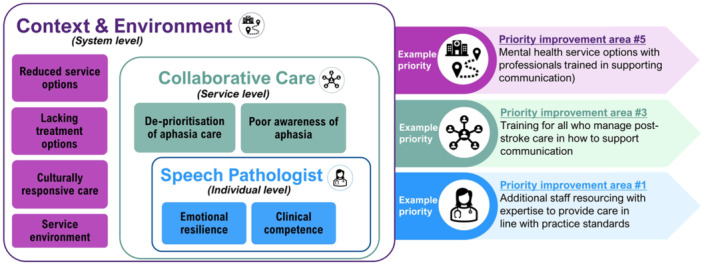
Touchpoints across journey of care delivery.

Three concentric levels of touchpoints were identified (Figure 5).

#### 3.4.1 Speech Pathologist

The speech pathologist's internal resilience, capabilities and professional support requirements were central to all aspects of care delivery. Emotional resilience was enhanced by access to professional supervision with experienced clinicians. Requiring support for decision making, brainstorming treatment approaches or opportunity to debrief with peers following emotionally complex case management was described. Speech pathologists reported needing access to professional expertise and continuing education to ensure clinical competence.

#### 3.4.2 Collaborative Care

Practitioners required speech pathology practices (language rehabilitation) to be valued within healthcare teams or service organisations (e.g., communication goals supported in length of hospital stay). Where aphasia care was de‐prioritised, this was described as de‐valuing the role of speech pathologists, limiting ability to offer care, or work collaboratively. Reduced awareness of aphasia among healthcare professionals reportedly decreased safety for people with aphasia (e.g., home visits to ensure communication safety before discharge not being supported) or management of procedural safety (e.g., communication strategies not taken on by treating teams delaying clinical assessments). Both de‐prioritisation of aphasia care and reduced awareness (of the impact of aphasia) was perceived to limit access to care and negatively influence health outcomes.

#### 3.4.3 Context and Environment

Reduced service options impacted continuity of care (e.g., lack of service providers to pick up care). Not having psychological services to provide care for people with aphasia, or lacking research evidence to inform clinical practice (e.g., treatment options for severe aphasia, or assessments identifying milder language deficits) caused concern and frustration. Participants described needing additional time and resources (e.g., flexible service options and interpreter services) to deliver equitable, culturally responsive care or research evidence detailing best‐practice across service contexts. Some service environments limited ability to deliver evidenced‐based care (e.g., lack of resourcing, time‐referenced services or no intensive rehabilitation options [remote sites]).

## 4 Discussion

The results presented explored the experiences of speech pathologists delivering aphasia care and their priorities for service design. Overwhelmingly, negative experiences described took place within hospital settings and related to the emotional toll of managing care. Reported experiences included lacking clinical supervision or peer support, and feeling unskilled or unprepared to make decisions about care, suggesting an alignment with prior research conducted both internationally and in Australia [10, 14, 33, 34, 35, 36]. Interestingly, those in this study highlighted a lack of control over care termination (all areas), and concern over causing harm (metropolitan only) if a patient was not ready to begin treatment. In prior research exploring clinicians' experiences ending treatment, Hersh [37] reported that clinicians felt torn coping with ending therapeutic relationships (i.e., internal conflict concerning decision‐making processes and how care termination was approached). Perhaps our reported lack of control over treatment decisions, and concern over causing harm, adds another layer to understanding the complexity of treatment endings and beginnings for speech pathologists. Time as a resource constraint was often associated with competing workload priorities [10, 14, 17], providing culturally responsive care [38, 39, 40], or mood management [17, 33, 41, 42] poststroke, mirroring prior findings. In this research, lack of time to provide culturally responsive care (e.g., rapport building/ensuring patient confidence, lacking standardised assessment tools or evidence‐based therapy options tailored for unique cultural needs) was additionally reported, irrespective of geographic remoteness, service context and seniority.

Priorities varied by geographic remoteness, with speech pathologists (regional/remote areas) valuing improved service linkages and support for patients transitioning to the community. Those in metropolitan areas felt dedicated aphasia staffing (speech pathologists) across stroke pathways was most essential. Given people with aphasia are at increased risk of complications during inpatient stays [43] and have poorer outcomes [43] it is perhaps not surprising that many priorities centred around dedicated upskilling of healthcare providers to support communication with a person with aphasia, and equitable, longer‐term access to aphasia treatment options. While priorities identified are somewhat aligned with previous research (e.g., identifying the top 10 research priorities specific to poststroke aphasia [44]), other priorities specific to aphasia service design also came through. For example, while speech pathologists described being cognisant of evidence‐based treatments, they reported needing further research to explore ways to implement and translate this evidence (often designed in metropolitan settings) into models of care specific to regional and remote service settings to facilitate implementation.

### 4.1 The Care Delivery Journey

This research sought to conceptualise the relationships between improvement areas across the care delivery journey for speech pathologists. Our findings, specific to delivering care for people with post‐stroke aphasia, in some ways parallel previous research reporting priories in broader poststroke care [45] (e.g., ‘health personnel's common understanding’ [*individual*‐level], ‘interaction and collaboration between health personnel [*service‐*level] and caseworkers across hospital and primary healthcare’ [*system‐*level]) [45]. Future service design (specific to enhancing aphasia care experiences) could be enhanced by exploring models of care in local contexts. However, improvement efforts targeting each level should be transferable to other contexts of hospital‐based or community‐based speech pathology practice (irrespective of geographic location). Service design should also consider implementing strategies targeting improvements across levels, given their concentric layering. For example, strategies targeting system‐level improvements only, might result in improving the environment (e.g., having an ideal space to delivery care), but without targeting the individual level, speech pathologists may still struggle to access the specialist expertise needed for improving patient experiences of care and outcomes.

### 4.2 Clinical Implications, Limitations and Conclusions

Interdisciplinary approaches might help to ensure communication is a focus of rehabilitation and more effectively supported for people with aphasia across disciplines. Collaborative practice provides opportunities for exposure to communication support techniques being used, a strategy thought to aid with the translation of new knowledge (e.g., communication support training) into practice [46]. Regular access to specialist clinical supervision, peer support and interprofessional practice may reduce organisational stress, support teams to deliver high‐quality aphasia care and provide opportunities for speech pathologists to upskill. Conceptualisation of the care delivery journey provides a blueprint for addressing core improvement areas in future aphasia service design, specific to speech pathologists.

A strength of this study was the diversity of experiences shared spanning geographic remoteness areas and service contexts across the care continuum. Study limitations include data collection during the COVID‐19 pandemic, where experiences shared may be less representative of usual aphasia care. Care should also be taken translating findings more widely within Australia or abroad.

This study offers novel insights into the challenges of speech pathologist's providing best‐practice aphasia services across the care continuum and areas of geographical diversity. Touchpoints and priorities identified provide a foundation for quality improvement in clinical practice. Future research should collaboratively combine priorities identified with those of people with aphasia and significant others. This is the second study in a program of research to establish a blueprint for improving poststroke aphasia services using EBCD.

## Author Contributions

**Lisa Anemaat**: conceptualisation, investigation, funding acquisition, writing–original draught, methodology, validation, visualisation, formal analysis, project administration, data curation, resources. **Victoria J. Palmer**: conceptualisation, writing–review and editing, visualisation, validation, methodology, supervision. **David A. Copland**: conceptualisation, funding acquisition, writing–review and editing, visualisation, validation, methodology, supervision. **Geoffrey Binge**: conceptualisation, methodology, writing–review and editing, supervision. **Kent Druery**: conceptualisation, methodology, writing–review and editing. **Julia Druery**: conceptualisation, methodology, writing–review and editing. **Kathryn Mainstone**: conceptualisation, methodology, writing–review and editing. **Bruce Aisthorpe**: conceptualisation, methodology, writing–review and editing. **Penelope Mainstone**: conceptualisation, methodology, writing–review and editing. **Sarah J. Wallace**: conceptualisation, investigation, funding acquisition, writing–review and editing, visualisation, methodology, validation, formal analysis, supervision.

## Ethics Statement

Ethical approvals were granted by the Royal Brisbane and Women's Hospital (HREC/2020/QRBW/61368) and The University of Queensland (2020000965) Human Research Ethics Committees.

## Consent

Consent was obtained using standard procedures.

## Conflicts of Interest

The authors declare no conflicts of interest.

## Supporting information

Supporting information.

## Data Availability

Anonymised data that support findings are available on request from the corresponding author (L.A.). Raw data are not publicly available to protect participant privacy.
