# Supplementary material for: Qualitative Exploration of Speech Pathologists' Experiences and Priorities for Aphasia Service Design: Initial Stage of an Experience‐Based Co‐Design Project to Improve Aphasia Services
Source: Health Expect. 2024 Jun 16;27(3):e14105. doi: 10.1111/hex.14105 (PMC11180296; doi:10.1111/hex.14105)
Supplement: Supplementary file 1 — Supporting information. [file HEX-27-e14105-s001.pdf]

Supplementary material

SM1. *Participating Hospital and Health Service Sites (n=21)*

Site	Hospital & Health Service (HHS)	HHS Districts	^Remoteness
1	Royal Brisbane and Women's Hospital	Metro North HHS	Major City
2	The Prince Charles Hospital	Metro North HHS	Major City
3	Redcliffe Hospital	Metro North HHS	Major City
4	Caboolture Hospital	Metro North HHS	Major City
5	Brighton Health Campus	Metro North HHS	Major City
6	*CBRT Caboolture	Metro North HHS	Major City
7	*CBRT North Lakes	Metro North HHS	Major City
8	*CBRT Chermside	Metro North HHS	Major City
9	*CBRT Redcliffe	Metro North HHS	Major City
10	Kilcoy Hospital	Metro North HHS	Inner Regional
11	Surgical Treatment & Rehabilitation Services	Metro North HHS	Major City
12	Toowoomba Hospital	Darling Downs HHS	Inner regional
13	Baillie-Henderson Hospital	Darling Downs HHS	Inner regional
14	Cairns Hospital	Cairns & Hinterland HHS	Outer Regional
15	Mount Isa Hospital	North West HHS	Remote
16	Longreach Hospital	Central West HHS	Very Remote
17	Barcaldine Hospital	Central West HHS	Very Remote
18	Sunshine Coast University Hospital	Sunshine Coast HHS	Major City
19	Gympie Hospital	Sunshine Coast HHS	Inner regional
20	Princess Alexandra Hospital	Metro South HHS	Major City
21	Gold Coast University Hospital	Gold Coast HHS	Major City
**Additional sites represented by speech pathologists responding to advertisements to be involved**			
**Site**	**Name of health service**	**HHS District operating in**	**Remoteness**
1	University student-led clinic – Mt Isa	North West HHS	Remote
2	Gidgee Healing – North West health district	North West HHS	Remote
3	Private practice – Toowoomba	Darling Downs HHS	Inner Regional
4	Community service – Atherton tablelands	Cairns & Hinterland HHS	Outer Regional
5	University student-led clinic – Toowoomba	Darling Downs HHS	Inner Regional

**CBRT: Community Based Rehabilitation Team*

^ *Remoteness determined by post-code using the Australian Bureau of Statistics Remoteness Area Classifications*

Notes: Service contexts included acute care, hyper-intensive stroke unit care, in-patient rehabilitation, community-based care, private practice, and university-based student-led clinics.

SM2. *Key touchpoints associated with positive experiences of care with example quotations*

Group	Example quotation
**Touchpoint 1 – Service and organisational factors influencing quality of care delivered**	
1.1 Ability to deliver comprehensive care	I think that was a good outcome in the end, um, even though that we did have those restrictions of that 12 weeks that we were kind of working with him, I feel like, um, we were able to achieve, you know, some good things in that time frame (SP-028-regional).
1.2 Personalising care and transition support – there's nowhere to go	…goal setting about what the patient actually wants, […] it made therapy functional for him, and it was actually motivating because he knew that we knew what he wanted, and we were working towards something for him (SP-013-regional).
1.3 Working in teams	…when you work together as a team and, um, pool all of your, um, professional knowledge and experience to be able to get some nice outcomes (SP-024- metropolitan).<br><br>I just remember being so excited when I could see this staff member now having a natural, normal, everyday conversation with this patient about who she was and what it meant to her and what her life experiences were, and it was only a 10-minute conversation but the significance it made to her [PWA], being recognized as a person, rather than just a patient […] made me feel worthwhile (SP-070-regional).
**Touchpoint 2 – Clinician factors impacting internal resilience and experiences of care delivery**	
2.1 Emotional toll for clinicians – I dreaded coming to work	…a patient I treated who was a 30-year-old woman who had had a stroke, um, delivering her first baby […] she hadn't met the baby yet, she was severely aphasic. I was able to use a multi-modal communication approach […] by teleconference and, um, meeting the bubby for the first time. So, that was, you know, it was huge for her. She was able to name the baby with – [….] with her family. […] really proud to be a – a speechie **(**SP-098-metropolitan).<br><br>…just seeing how speech pathologists can make a difference […] just so rewarding when you are able to see a difference that, um, the service is making for patients (SP-054- metropolitan).<br><br>…it made me really grateful to think that, as part of my career, I've been able to do that for someone (SP-036-metropolitan).
2.3. Knowing care delivered was meaningful	…you can do an hour of impairment-based therapy a day, but what really mattered most for her was that half an hour supported conversation with a loved one (SP-002-regional).<br><br>…there is that moment, when you get, like, goosebumps because you've done something with a patient, or a patient has given you feedback that what you said and what you've helped them to do has been so meaningful in their lives (SP-093a- metropolitan)<br><br>"then we'd walk around her garden, and she would tell me the names of the different plants" (SP-022-metropolitan)<br><br>…the relief on her face and the excitement that she had […] to share that with her in a more functional way that wasn't just come and sit in a room and repeat words over and over again" (SP-071-regional).
**Touchpoint 3 – Patient factors influencing care management, delivery, and outcomes**	
3.1 Good outcomes and communication successes	…we were working on a goal of talking on the phone, […] she came into a therapy session, one day, and just told me, she said, 'I spoke on the phone with my sister for the first time in 12 years,' and it was such a nice moment for her that she was able to achieve that (SP-041- metropolitan).
3.2 Patient engagement and motivation	I think it was positive for me because he actually wanted to be there, and he actually wanted to do it, and we did see a positive outcome (SP-017-remote).
3.3 People with aphasia feel heard and acknowledged	I think there was a breakthrough moment for all of us, where he- he wanted to select a type of coffee with how many sugars and he got it and that relief that he got, um, from saying, 'actually, I just want those two sugars in' […] the relief you saw on him when he could get that information out (SP-031-regional)<br><br>I think was just really meaningful for both of us and really, like it made me very happy at the end that we had gotten there and to actually see her leave a session not in tears and smiling. Um, and for her to actually be able to, I guess, share something meaningful (SP-071-regional)
**Touchpoint 4 – External and environmental factors impacting care**	
4.1 Family engagement – it's not just the patient but support around them	…the other part that made it successful was that he had a really, um, amazing partner who really advocated for him, as well, and supported him through that process […] really enabled him to be able to get home (SP-100-metropolitan).
4.4 COVID - Modification of care during a pandemic	…whatever I did was an extension of, um, communicating, you know, the physio home programme, the occupational therapy home programme, supporting the rehab consultant with what she had recommended. […] the whole programme was run over telehealth through the interpreter and [we] worked very closely with the interpreter, like pre-briefing, debriefing, um, in order to try to get it to be as accurate as possible. […] it could have gone wrong in so many places, and yet I feel like we were able to deliver the maximum value that we possibly could (SP-008-metropolitan).

SM3. *Summary of leading local rankings identified speech pathologists across geographic remoteness*

Priority ID	Themed priority domain	Overall rank	Geographic remoteness		
			REM	REG	MET
**Priority 31**	31.Additional staff resourcing with suitable expertise is needed in stroke units, acute, rehabilitation and community service settings to facilitate provision of quality care in line with practice standards for people with aphasia. This includes speech pathology staff rotations to remote locations to provide work cover and to improve understanding of remote service model contexts within the profession.	#1	#5		#1
**Priority 34**	34. Service models that cater for in-patient and out-patient hospital services to be able to conduct sessions within the home or community environment with a person with aphasia. This includes allowing a patient to receive in-patient services within their home or community context at the same intensity (for the duration of their sub-acute rehabilitation stay).	#2			#2
**Priority 9**	9. Regular delivery of **communication partner training** for all people involved with managing the care of a person with aphasia and their families is needed. This includes improved access to **standardised training resources**.	#3		#4	#4
**Priority 29**	29. Improved **access to intensive communication therapy options**, with increased frequency of services available per day, for all people with aphasia **across geographic remoteness** settings. This includes access to **regular therapy blocks** for people with aphasia **after returning to the community with ongoing availability**. Services should also have a focus on combining both functional and impairment-based approaches.	#4			#3
**Priority 27**	**27. Mental health service options** and support for PWA and family across the continuum. This includes mental health and social work **professionals who are trained in supporting communication** and co-facilitated team approaches to ensure communication is able to be supported.	#5			
**Priority 19**	19. Improved access to **longer-term and ongoing services** and knowledge of **available service options** for people with aphasia is needed. This includes **review clinics**, state-wide databases of patients for follow up and **dedicated service options for those living in the community** to access as needed.				#5
**Priority 33**	**33. Financial resourcing** to facilitate provision of **evidenced based care** for people with aphasia **in RACF and regional and remote locations**. This includes access to **stroke team expertise**, support to provide **care closer to home** in rural locations and access to resources to advocate for funding.		#2	#3=	
**Priority 20**	20. Increased **support for** people with aphasia **transitioning** to the community or between services. This includes resources to facilitate education delivery (for healthcare staff and patients) on **referral pathways** and how to access **on-going support** and supporting PWA with **linking to suitable services**.			#1	
**Priority 16**	**16. Resources to support care delivery** - Access to **ready to use therapy resources**, up to date research, and advice. This includes **demonstrational videos** that are available online.			#2	
**Priority 28**	28. Collaborative **inter-professional goal setting, therapy planning** and **treatment approaches** that are patient and family centred and **adapt to patients needs**. This includes dedicated goal setting resources and student training to support personalised goal setting.		#4		
**Priority 13**	**13. Education** for people with aphasia and their significant others that **supports transitioning to the community** and ways to track delivery of education across multiple stages of recovery.		#1=		
**Priority 21**	**21. Comprehensive pathway information** and **resources describing** the **journey of care** to enable delivery of increased support for people with aphasia **transitioning to the community**.			#3=	
**Priority 4**	4. Community and health service **awareness of communication disability** (eg: aphasia) and **how to support communication**.			#5	
**Priority 18**	**18. Research** topic areas (**assessment norms for PWA 80+years** and **how to measure outcomes at end of life**), administration processes and supported **knowledge translation of new research**. This includes improved processes to **support PWA to access research participation** opportunities and **translation of research therapy approaches** for effective delivery in **remote service settings.**		#3		
**Priority 7**	**7.** Improved **understanding of how culture impacts care management and patient engagement** for Aboriginal and/or Torres Strait Islander peoples with aphasia and development of **culturally appropriate ways to manage care.**		#1=		
